# Tribute to Alberto J. Kaumann

**DOI:** 10.1007/s00210-025-04708-5

**Published:** 2025-10-18

**Authors:** Torsten Christ, Ursula Ravens

**Affiliations:** 1 Institut für Experimentelle Pharmakologie und Toxikologie, Universitätsklinikum Hamburg-Eppendorf, Martinistraße 52, 20246 Hamburg, Germany; 2 Institut für Experimentelle Kardiovaskuläre Medizin, Universitätsklinikum Freiburg, Elsässerstrasse 4C, 79110 Freiburg, Germany

**Keywords:** Cardiovascular adrenergic and serotonergic systems, β-adrenoceptors, cAMP

## Abstract

This review summarises the major contributions of Alberto J. Kaumann who died in December 2024. The German-born pharmacologist devoted his scientific life to the cardiovascular adrenergic and serotinergic systems. He classified the subtypes of the cardiac β-adrenoceptors (β-AR) into β_1_- and β_2_-AR using the subtype-selective antagonist. In addition, he showed that the dual coupling of β_2_-AR to Gα_s_- and Gα_i_-proteins plays a minor role in the healthy heart. He also found that the positive inotropic effect of serotonin (5-HT) was not mediated by release of noradrenaline, but due to activation of a specific 5-HT receptor coupled to Gα_s_-proteins. His experiments with prostaglandin-E_1_ demonstrated an increase in cAMP and spontaneous beating frequency of the heart in the absence of a positive inotropic effect, suggesting a compartmentation of cAMP. This finding was later verified by experiments with subtype-selective phosphodiesterase inhibitors. Last not least, he explained the antiarrhythmic effect of sotalol by prolongation of the cardiac action potential duration, providing for the first time, what was years later to be defined as class III antiarrhythmic action. With Alberto Kaumann, we have lost a colleague and friend who had dedicated his life to science and music.

Alberto Julio Kaumann, a lone wolf in Pharmacology, died on December 26, 2024, in Alicante (Spain) at the home of his son. With him, we lose a dedicated, headstrong colleague who was not easily impressed by any new trendy research directions but pursued his own ideas with great consequence and perseverance. He loved the beauty of experimental work and science. His first published paper “Heart rate acceleration by imipramine and by noradrenaline after imipramine; its blockade by dichloroisoproterenol (DCL)” (Kaumann et al. 1962) reads like a revelation of his life-long scientific passion.

Born into an old Jewish merchant family in Hamburg, he had to leave Germany as a small boy together with his mother and younger brother in order to find refuge in Buenos Aires. In the turmoil of post-war times, his mother disappeared and left him to care for his brother. Besides making a living by delivering letters and catching frogs, which he sold to restaurants, he was eager to learn whatever there was to know. He made it into Medical School in Buenos Aires and joined the Department of Pharmacology while still a student. It was here that he discovered his love for science and therefore went on to one of the best Pharmacological Departments in the USA, at Harvard Medical School. He returned to Argentina with the intention of pursuing a career in Pharmacology. However, the political turmoil forced him to leave his country. Luckily, he found refuge and a new scientific habitat upon invitation of the late Physiologist Raimund Kaufmann at the Institute of Clinical Physiology, University of Düsseldorf. During this period, Alberto frequently travelled to East Germany to work with Albert Wollenberger, the inventor of the famous Wollenberger-clamp for rapid freezing of myocardial tissue samples (Wollenberger et al. 1960). Eventually, the Nobel prize winner James Black persuaded Alberto to join Imperial Chemical Industries (ICI), where he stayed until retirement. Although he had his official scientific home in the Department of Physiology at Cambridge University, his retirement marks the beginning of scientific wandering around the world, spending various periods of time in the laboratories of scientific friends in Oslo, Brisbane, Dresden, and Murcia. As frailty because of old age increased, he was taken care of by his son in Alicante.

## Scientific achievements

**Fig. 1:**
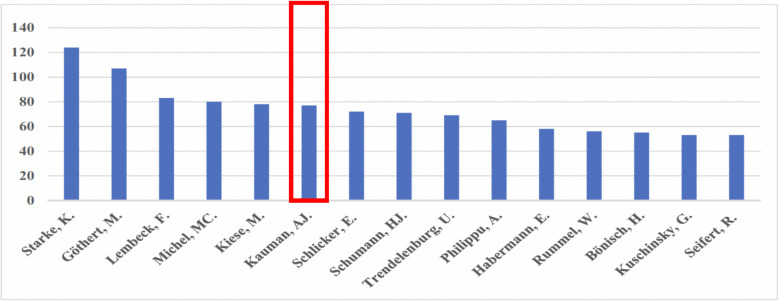
Top 15 contributors to Naunyn–Schmiedeberg’s Archives of Pharmacology. Red box marks position 6 for A. J. Kaumann. Adapted from Dats et al. (2023), with permission of the publisher

Right from his very first encounter with science, Alberto became fascinated by the cardiovascular adrenergic and serotoninergic systems to which he devoted his entire scientific oeuvre. Independent in his thinking and always sceptical about results from experiments he did not conduct himself, he has nevertheless published 206 scientific papers, of which 79 appeared in Naunyn–Schmiedeberg’s Archives of Pharmacology. In fact, he holds rank six among the 15 most cited authors in our journal (Fig. 1; Dats et al. 2023).

### Noradrenaline, adrenaline, and adrenoceptors

The large family of G-protein-coupled receptors (GPCR) includes receptors activated by sympathomimetic amines (adrenoreceptors, AR) that have been classified into α- and β-subgroups based on differences in responses observed in various organs (for review, see Ahlquist 1980). Further division of the β-AR subgroup was suggested to explain the different orders of potencies of various chemically modified agonists with respect to cardiostimulatory and bronchodilatory effects (Lands and Brown 1964). The cardiostimulatory receptor was designated β_1_-AR. The receptor mediating all other adaptations to acute stress, such as vascular and bronchial smooth muscle relaxation, increase in skeletal muscle strength, and glycogenolysis, was called β_2_-AR. The discrimination was rather simple, since noradrenaline preferentially activates β_1_-AR and is devoid of action on non-cardiac tissues, whereas adrenaline is considered to affect non-cardiac tissue via β_2_-AR. The well-known cardiostimulant effect of adrenaline could be easily explained by adrenaline’s ability to activate both β_1_- and β_2_-AR.

The final distinction between β_1_- and β_2_-AR became evident with the availability of new β-subtype-selective antagonists (Barrett et al. 1968). These compounds differentially attenuated the positive chronotropic effects of noradrenaline and adrenaline in cat right atria: While the unselective β-AR antagonist propranolol blocked the positive chronotropic effects of both noradrenaline and adrenaline, the selective β_1_-AR antagonist practolol only affected the response to noradrenaline, and the β_2_-AR antagonists H 35/15 that to adrenaline (Carlsson et al. 1972). These findings strongly suggested the existence of both β_1_- and β_2_-AR in the myocardium, which was later confirmed by radioligand binding studies (Stiles et al. 1983; Brodde et al. 1983) and cloning the receptors from human heart tissue (Frielle et al. 1987; Kobilka et al. 1987).

The discovery of β_2_-AR in the human heart appeared to offer a novel strategy for the treatment of heart failure: selective β_2_-AR agonists were expected to dilate coronary vessels and at the same time enhance force development. The latter effect seemed particularly useful since β_2_-AR was neither desensitized nor down-regulated in human heart failure (Bristow et al. 1986). Of the many groups working on this topic, it was Alberto Kaumann who demonstrated that in the human heart, selective β_2_-AR stimulation was associated with the disastrous side effects of catecholamines: the induction of arrhythmias (Kaumann and Sanders 1993).

Reliable pharmacological characterization of β-AR subtypes was hampered by the limited selectivity of either activating or inhibiting ligands; some of the antagonists even showed the phenomenon of intrinsic activity. For his entire remaining career, Alberto tackled these difficulties by consistently using the natural catecholamines for selective β_1_- and β_2_-AR activation: noradrenaline in the presence of the most selective β_2_-AR antagonists available, ICI 118,551, and adrenaline in the presence of the β_1_-AR antagonist CGP20712A. By this approach, he was able to dissect precisely β_2_-AR-dependent actions even in animals initially believed to be regulated purely by β_1_-AR (Christ et al. 2009).

Alberto has always been sceptical about universal truths. Thus, he described in much detail the apparently paradoxical effects of adrenaline in mice with cardiac overexpression of the human β_2_-AR. These findings suggested that β_2_-AR can couple both to Gα_s_ and Gα_i_ proteins (Heubach et al. 2003, 2004). Alberto spent considerable energy to emphasize that such effects probably result from the artificial overexpression that is not representative of the *healthy* human heart (Molenaar et al. 2007), although dual coupling of β_2_-AR to Gα_s_ and Gα_i_ had been shown before in ventricular cardiomyocytes from patients with severe *heart failure* (del Monte et al. 1993).

These early observations of differential effects of the two natural catecholamines noradrenaline and adrenaline by Alberto and his colleagues paved the way for even more sophisticated distinctions. Today, many new facets of signal transduction via cardiac adrenoceptors have been elucidated. One example is the concept of “biased” signaling by specific compounds, i.e., the preferential activation of one downstream pathway over the other when a receptor is coupled to more than a single G protein or signaling cascade (Ma et al. 2025). Inhibitors of G-protein-coupled receptor kinases, which prevent desensitization of β-AR upon prolonged stimulation, modulate the inotropic responses (Brand et al. 2024). In addition, recent evidence suggests that even within the same (murine) heart, individual cardiomyocytes do not express a constant pattern of β- and α-AR, but at least four distinct cardiomyocyte phenotypes with various patterns have been found (Myagmar et al. 2017).

### Serotonin

**Fig. 2:**
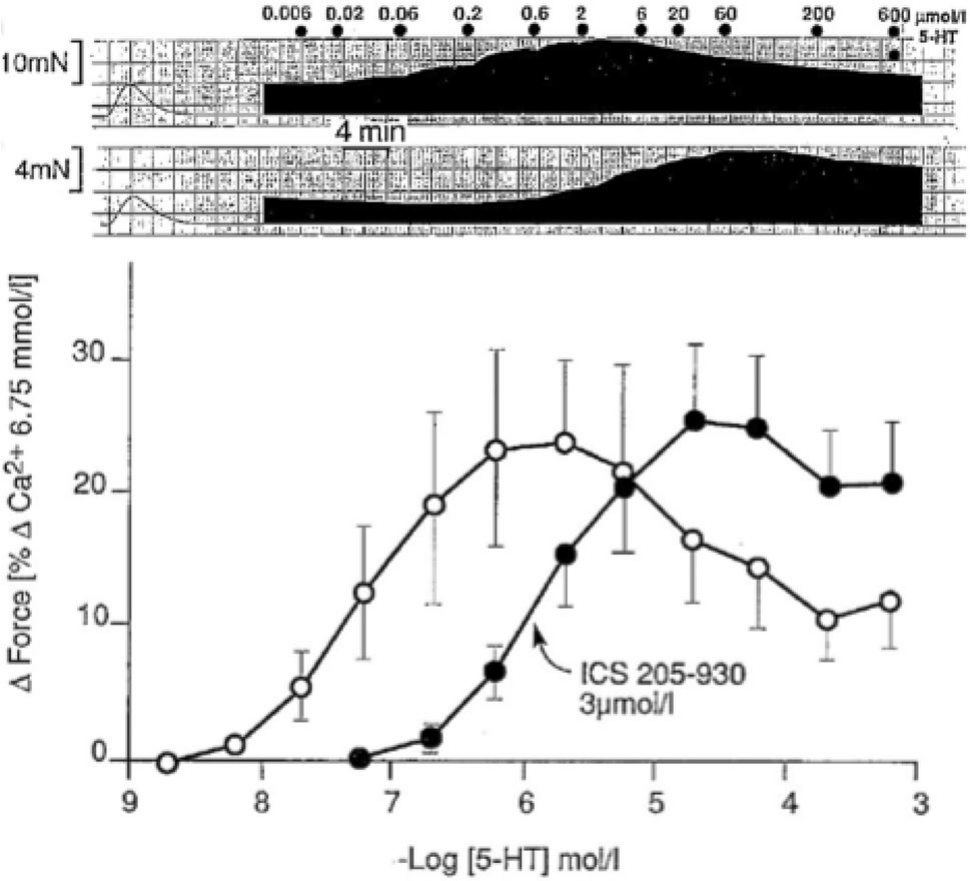
Positive inotropic effect of serotonin in human left atrial preparations. Above: force of contraction (frequency of electrical stimulation 0.5 Hz) with increasing concentrations (in µM) of serotonin under control conditions (upper trace) and in the presence of the 5-HT receptor antagonist ICS 205–930 (tropisetron, 3 µM). Below: concentration–response curves for the above experiments (mean ± S.E.M, *n* = 6). From Sanders and Kaumann (1992) (with permission of the publisher)

Many cardiovascular pharmacologists will associate the name of Alberto Kaumann with serotonin (e.g., Kaumann 1983, 1990; Kaumann et al. 1994). The positive inotropic effect of serotonin (5-HT) has been known since the 1960s (Trendelenburg 1960) and was interpreted as serotonin-induced release of noradrenaline from intracardiac nerve endings. Inspired by Halima Ouadid (Ouadid et al. 1992), Alberto discovered that the 5-HT-induced enhancement of contractile force could be prevented and/or reversed by 5-HT receptor antagonists even when myocardial noradrenaline stores were emptied (Sanders and Kaumann 1992). Later, he could show that in the human heart, 5-HT receptor expression is not restricted to the atrium (Fig.2) (Kaumann and Levy 2006).

### Action potential-prolonging effect of the β-adrenoceptor antagonist sotalol

**Fig. 3:**
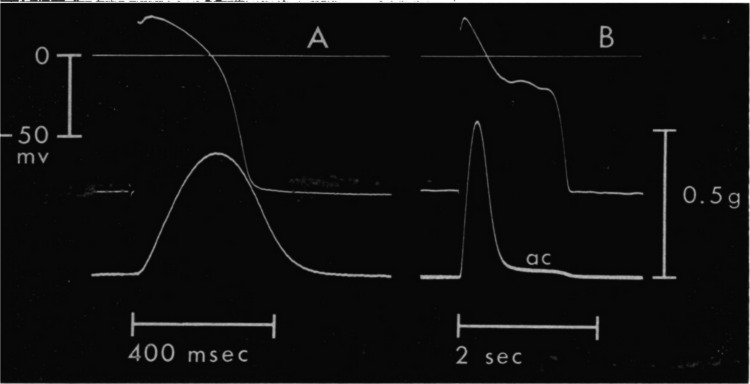
Action potentials (upper traces) and force of contraction (lower traces) of a kitten papillary muscle (stimulation frequency 0.2 Hz, 32.0–32.5 °C). **A** pre-drug control, **B** in the presence of sotalol (6 × 10^−^.^4^ M, 30 min). Please note the different time scales in (**A**) and (**B**). From Kaumann and Olson (1968) (with permission of the publisher)

Less well-known are Alberto’s contributions to understanding the antiarrhythmic action of sotalol. Well before Vaughan Williams (Vaughan Williams 1975) suggested that prolongation of the cardiac action potential is an antiarrhythmic principle (class III action), Alberto wondered why the β-adrenoceptor antagonist sotalol had a positive inotropic effect despite the simultaneous presence of the standard β-adrenoceptor antagonist propranolol. In fact, sotalol increased isometric contraction amplitude in feline papillary muscle and even induced a small, but persisting aftercontraction. He postulated that this behavior could be due to a prolongation of the electrical excitation. It took him quite some persuasive effort to convince his colleague Camille Olson that action potential recordings would provide an answer to this riddle. The seminal observation of an enormously prolonged action potential duration after exposure to an admittedly extremely high concentration of sotalol (see Fig. 3) was published in Alberto’s first Science paper (Kaumann and Olson 1968).

### Compartmentation of cAMP

**Fig. 4:**
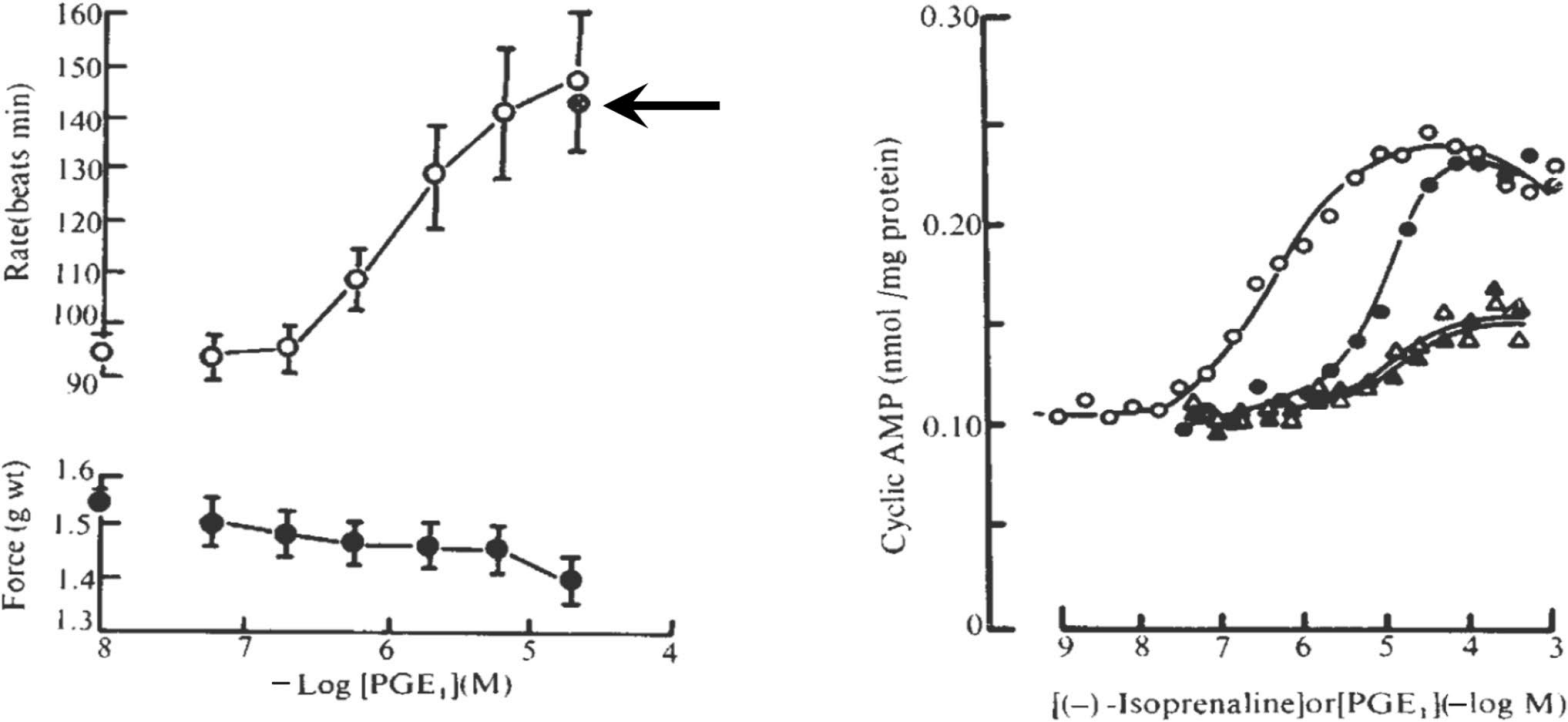
Left: PGE_1_ effects on intact atria: chronotropic effects in spontaneously beating right atria (top) and inotropic effects (bottom). Data for PGE_1_ in the presence of 100 nM propranolol are marked by an arrow. Right: Effects of (−)-isoprenaline (circles) and PGE_1_ (triangles) in the absence (open symbols) or presence (closed symbols) of 100 nM propranolol on cAMP in membrane particles of atria from kittens. From Kaumann and Birnbaumer (1974) (with permission of the publisher)

To the best of our knowledge, Kaumann and Birnbaumer (Kaumann and Birnbaumer 1974) provided the first evidence for compartmentation of cAMP, without coining the term as such. Based on similarities of chemical structural elements between catecholamines and prostaglandin-E_1_ (PG-E_1_), they investigated whether the two compounds would share a common receptor. They observed that PG-E_1_ produced a positive chronotropic effect in spontaneously beating kitten atria, that was not antagonised by propranolol, yet failed to enhance force of contraction despite a pronounced increase of cAMP in membranes from atrial and ventricular myocardium (Fig. 4). Thus, they could exclude the involvement of β-adrenoceptors in the cAMP increase and, at the same time, propose that there was a site of cAMP production that was not relevant for the force of contraction. Incidentally, cAMP production in response to 5-HT_4_ receptor stimulation was diminished in remodeled atrial tissue from patients with persistent atrial fibrillation, and so were the positive inotropic and arrhythmogenic effects (Christ et al. 2014). The reduced cAMP production and positive inotropic effect were rescued when both cAMP degrading enzymes phosphodiesterase 3 and 4 were inhibited, but the low arrhythmic response remained unaffected by the enzyme inhibition (Berk et al. 2016). These results were also interpreted as compartmentation of cAMP production and have been published in Alberto’s very last paper (Dolce et al. 2021).

**Fig. 5:**
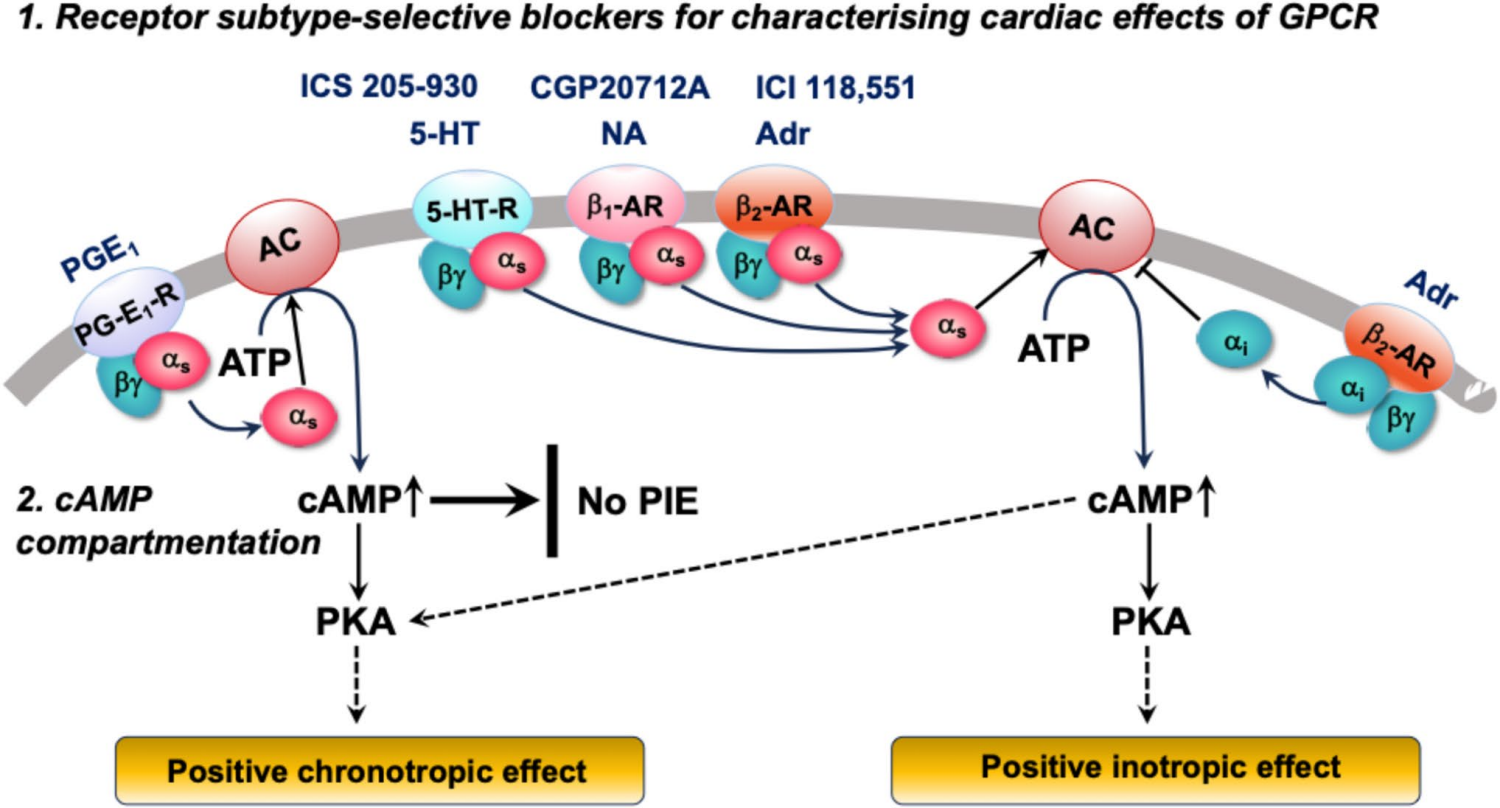
Schematic of two major contributions by Alberto Kaumann. The cardiac GPCR with the highest binding affinity for prostaglandin-E_1_ (PGE_1_), serotonin (5-HT), noradrenaline (NA), and adrenaline (Adr) are PGE_1_-R, 5-HT_4_-R, β_1_-AR, and β_2_-AR, respectively. All of them couple to Gα_s_ proteins, stimulate cAMP production, and increase spontaneous beating rate, but produce differential positive inotropic effects (PIE), suggesting compartmentation of cAMP production. Please note that activation of Gα_i_ by β_2_-AR stimulation is restricted to mice overexpressing β_2_-AR

Alberto Kaumann dissected the often puzzling and controversial effects of GPCR stimulation with the help of receptor subtype-selective antagonists and by careful observation of different functional parameters such as force of contraction, spontaneous beating frequency, and cAMP generation. Thus, he could differentiate the functional consequences of β_2_-AR coupling to Gα_s_ and Gα_i_ proteins and provide the first evidence for cAMP compartmentation in responses to various GPCR stimulations (Fig. 5).

## Concluding remarks about a special personality

**Fig. 6:**
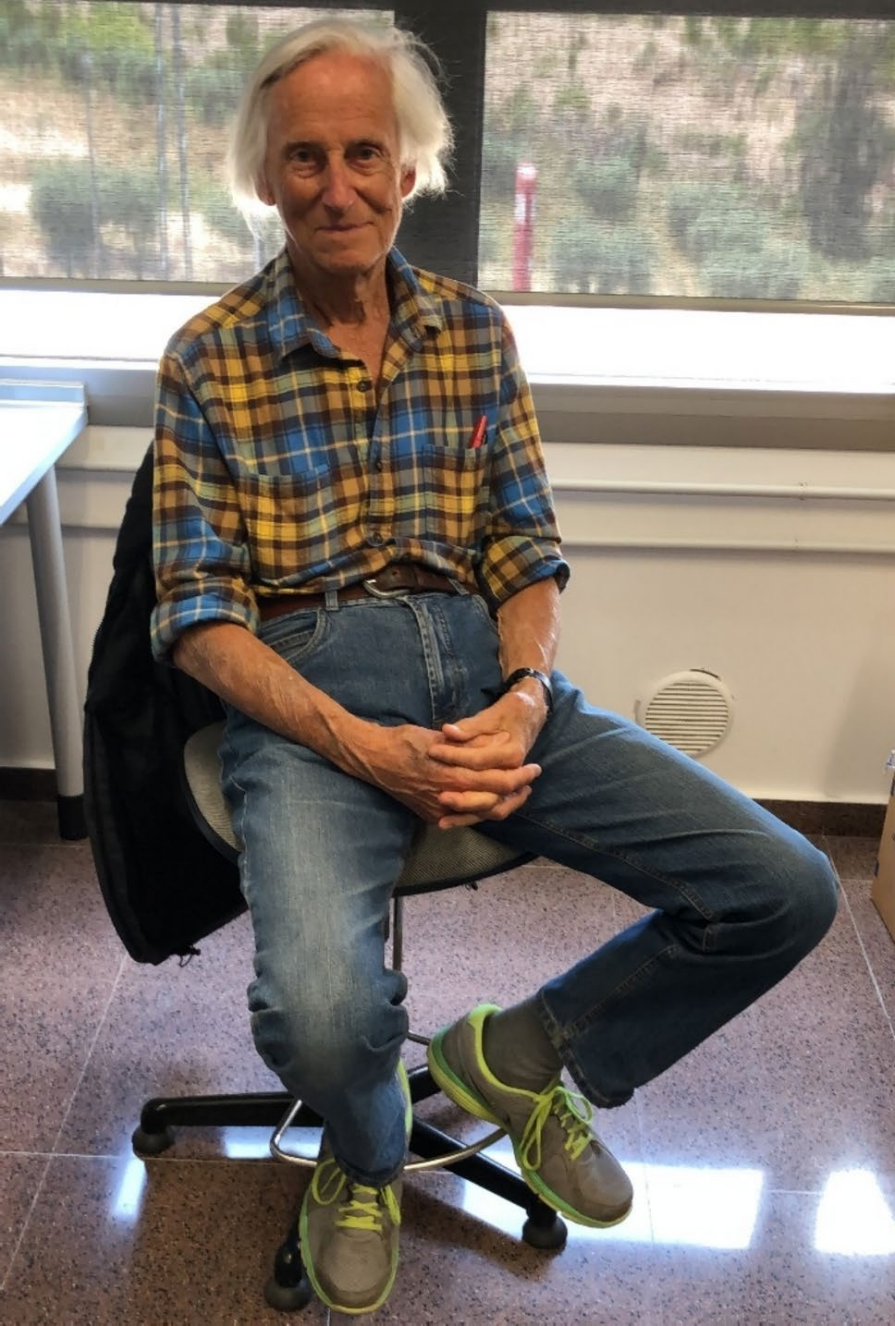
Alberto Kaumann in the Department of Pharmacology at the University of Murcia, Murcia, Spain (by courtesy of Dr. Alejandro Galindo-Tovar)

Alberto Kaumann led a modest daily life (Fig. 6), but had the highest demands on himself and others concerning science and music. He boundlessly admired Johann Sebastian Bach’s music for its precision and genial musicality, and was able to factor out Bach’s known antisemitism. He was a passionate piano player, practicing every day for an hour or two. Not only did he love to play for himself, but also for colleagues or—when in Dresden—even for residents of an old people’s home.

In an interview, one of the authors (UR) described Alberto Kaumann’s impact on pharmacology as “a fine example of how simple experimental methods, like measuring force of contraction in isolated atrial muscle strips, are still useful for resolving pharmacological problems if the results are interpreted by a brilliant mind” (Circulation 2010: Spotlight in Cardiology). We, the authors, share with Alberto what he called the “libido” for science and music.

## Data Availability

All source data for this work (or generated in this study) are available upon reasonable request.
